# Early postnatal cardiac manifestations are associated with perinatal brain injury in preterm infants with twin to twin transfusion syndrome

**DOI:** 10.1038/s41598-019-54951-z

**Published:** 2019-12-06

**Authors:** Hannah Cho, Seung Han Shin, Jong Kwan Jun, Seung Hyun Shin, Yoo-Jin Kim, Seh Hyun Kim, Ee-Kyung Kim, Han-Suk Kim

**Affiliations:** 1 Seoul National University College of Medicine, Department of Pediatrics, Seoul, 110-769 Republic of Korea; 2 Seoul National University College of Medicine, Department of Obstetrics & Gynecology, Seoul, 110-769 Republic of Korea

**Keywords:** Cardiovascular diseases, Neurological disorders, Paediatric research

## Abstract

Altered hemodynamics associated with twin to twin transfusion syndrome (TTTS) can be manifested in the fetal and neonatal heart. This study evaluated the association between cardiac manifestations immediately after birth and brain injury in preterm infants with TTTS. Medical records of preterm infants who were born at <35 weeks of gestation with TTTS and admitted to the neonatal intensive care unit at Seoul National University Children’s Hospital between January 2011 and January 2018 were reviewed. TTTS was prenatally diagnosed and staged according to the Quintero criteria. Echocardiographic findings, brain ultrasound and MRI imaging findings were analyzed. Fifty-three infants were enrolled in this study. Thirty-two infants (60.3%) were treated by fetoscopic laser coagulation. Brain injury developed in 15 infants (28.3%). Hypotension within the first week and immediate postnatal cardiac manifestations were more prevalent in the brain injury group. In the multivariate analysis, acute kidney injury and cardiac manifestations, such as ventricular dysfunction and tricuspid regurgitation, were statistically associated with brain injury in the study population. Immediate postnatal cardiac manifestations, such as ventricular dysfunction and tricuspid regurgitation, can serve as surrogate markers for perinatal hemodynamic disturbance, which are associated with early neonatal brain injury in preterm infants with TTTS.

## Introduction

Twin to twin transfusion syndrome (TTTS) occurs in 10 to 15% of monochorionic twin gestations^1^. The pathophysiology of TTTS is intertwin blood transfusion through a placental vascular anastomosis^2^. As a result of hemodynamic imbalance and prematurity, newborns with TTTS experience various comorbidities and high mortality^3^.

Brain injury is an important comorbidity associated with long-term sequelae in TTTS. The incidence of brain injury in TTTS is 3~41%^4–6^. Low gestational age at birth is an important risk factor for long-term neurodevelopmental impairment in TTTS treated with fetoscopic laser surgery^7^. However, risk factors for perinatal brain injury in TTTS have not been clearly elucidated. A prospective study comparing TTTS and dichorionic twin neonates reported that only gestational age was a significant risk factor for severe cerebral lesions^8^. Several twin studies have implicated intrauterine hemodynamic disturbances as risk factors for brain injury in TTTS. In a systematic review of several monochorionic twin studies, abnormal umbilical artery Doppler findings and larger twins were associated with severe cerebral injury^9^.

TTTS is recognized as a cardiovascular disease of twins based on unequal circulating volumes with hypervolemia in the recipient and hypovolemia in the donor fetus. As a result, functional cardiovascular changes can be manifested starting in the early stage of TTTS^10^. These changes can further lead to the development of structural heart disease, which may present as various cardiac manifestations before and immediately after birth.

Because hemodynamic disturbance is an important pathophysiology of both brain injury and the cardiac manifestations of TTTS, it is possible that cardiac manifestations immediately after birth may be associated with brain injury in either the donor or the recipient in TTTS. The aim of this study was to evaluate the association between cardiac manifestations immediately after birth and brain injury in preterm infants with TTTS.

## Results

During the study period, 53 infants were diagnosed with TTTS in utero and born before 35 weeks of gestation. In terms of Quintero staging, 6 (11.3%) infants were stage I, 2 (3.8%) were stage II, 21 (39.6%) were stage III, 16 (30.2%) were stage IV, and 8 (15.1%) were stage V. Thirty-two infants (60.3%) were treated by fetoscopic laser coagulation.

Birth weight was lower in the donor group, and small for gestational age (SGA) and acute kidney injury (AKI) were more prevalent in the recipient group (Table 1). Neonatal morbidities, such as respiratory distress syndrome (RDS), bronchopulmonary dysplasia (BPD), treated patent ductus arteriosus (PDA), necrotizing enterocolitis (NEC) and sepsis, were comparable between the two groups. However, cardiac manifestations, such as ventricular dysfunction, ventricular hypertrophy, tricuspid regurgitation (TR) and mitral regurgitation (MR), were more prevalent in the recipient group (Table 2). There was no difference in brain injury between the two groups.

**Table 1:** Demographics of TTTS donors and recipients.

	Donor (n = 23)	Recipient (n = 30)	p value
Gestational age (week)	30 (27.9–31.1)	30.4 (27.9–31.4)	0.733
Birth weight (g)	950 (770–1250)	1580 (1160–2010)	<0.001
Small for gestational age	13 (56.5)	4 (13.8)	0.002
Male	10 (43.5)	13 (43.3)	1.000
Cesarean section	18 (78.3)	20 (66.7)	0.539
Apgar score 1 minute	4 (2–6)	4 (2–6)	0.828
Apgar score 5 minute	6 (5–7)	7 (6–7)	0.350
Initial hemoglobin (mg/dL)	15.2 (14.2–18.6)	14.9 (12.9–18.2)	0.733
Hypotension	6 (27.3)	10 (33.3)	0.765
Respiratory distress syndrome	12 (52.2)	16 (53.3)	1.000
Moderate to severe bronchopulmonary dysplasia	8 (34.8)	6 (20.0)	0.346
Treated patent ductus arteriosus	11 (47.8)	9 (30.0)	0.255
Maximum creatinine (mg/dl)	1.08 (0.89–1.84)	1.01 (0.89–1.4)	0.270
Acute kidney injury	7 (30.4)	2 (6.7)	0.031
Necrotizing enterocolitis	1 (4.6)	3 (10.0)	0.629
Sepsis	4 (17.4)	2 (6.7)	0.385
Retinopathy of prematurity operation	1 (4.4)	0 (0)	0.434
Mortality	4 (17.4)	4 (13.3)	0.715

Values are expressed N (%) or median (interquartile range).

**Table 2:** Cardiac manifestations and brain injury in TTTS donors and recipients.

	Donor (n = 23)	Recipient (n = 30)	p value
Cardiac manifestation	11 (47.8)	26 (86.7)	0.006
Pulmonary hypertension	8 (34.8)	15 (50.0)	0.402
Ventricular dysfunction	1 (4.4)	13 (44.8)	0.001
Ventricular hypertrophy	5 (21.7)	14 (48.3)	0.081
Interventricular septal deviation	4 (17.4)	7 (24.1)	0.735
Right to left shunt	3 (13.0)	4 (13.8)	1.000
Tricuspid regurgitation	1 (4.4)	9 (32.1)	0.015
Mitral regurgitation	0 (0)	7 (24.1)	0.013
Left ventricular outflow tract stenosis	0 (0)	1 (3.5)	1.000
Right ventricular outflow tract stenosis	0 (0)	4 (13.8)	0.120
Brain injury	4 (17.4)	11 (36.7)	0.140
Hypoxic injury	1 (4.4)	3 (10.0)	0.624
Intraventricular hemorrhage ≥ Grade 3	0 (0)	1 (3.3)	1.000
Infarction	1 (4.4)	7 (23.3)	0.118
Periventricular leukomalacia	2 (8.7)	9 (30.0)	0.089
Atrophy	0 (0)	1 (3.3)	1.000

Values are expressed N (%) or median (interquartile range).

Brain injury developed in 15 (28.3%) infants (Table 3). Maximum creatinine during the first week was higher in the brain injury group (p < 0.001), and the incidence of AKI during the first week was slightly higher in the brain injury group, without statistical significance (p = 0.097). Early postnatal hypotension was also more prevalent in the brain injury group (p = 0.044). The distributions of Quintero stage and treatment modality for TTTS did not differ between the brain injury group and the no brain injury group (Table 3). There were no differences in the timing of TTTS treatment and the interval from treatment to birth between the two groups. Overall cardiac manifestations (60.5% vs. 93.3%, p = 0.022) and findings, such as ventricular dysfunction (13.5% vs. 60.0%, p = 0.001) and TR (10.8% vs. 42.9%, p = 0.018), were more prevalent in the brain injury group.

**Table 3:** Baseline characteristics of the brain injury group and the no brain injury group.

	No brain injury (n = 38)	Brain injury (n = 15)	p value
Gestational age (week)	30.1 (27.9–31.9)	30.6 (27.9–31.1)	0.859
Birth weight (g)	1195 (900–1490)	1500 (880–1930)	0.260
Recipient	19 (50)	11 (73.3)	0.140
Male	17 (44.7)	6 (40)	1.000
Small for gestational age	14 (36.8)	3 (21.4)	0.127
Cesarean section	27 (71.1)	11 (73.3)	1.000
Initial hemoglobin (mg/dl)	15.2 (13.1–17.1)	14.7 (13.3–20.4)	0.636
Apgar score 1 minute	4 (2–6)	3 (2–5)	0.449
Apgar score 5 minute	7 (5–7)	6 (5–7)	0.290
Respiratory distress syndrome	19 (50)	9 (60)	0.556
Treated patent ductus arteriosus	19 (41.3)	12 (46.2)	0.805
Maximum creatinine (mg/dl)	0.95 (0.79–1.29)	1.5 (1.3–1.9)	<0.001
Acute kidney injury	4 (10.5)	5 (33.3)	0.097
Hypotension	8 (21.6)	8 (53.3)	0.044
Quintero stage ≥4	17 (44.7)	7 (46.7)	1.000
TTTS treatment			0.475
Amnioreduction	11 (29)	2 (13.3)	
Fetoscopic laser coagulation	22 (57.9)	10 (66.7)	
Gestational age at treatment (week)	23.1 (21.1–25.4)	22.4 (21–24.4)	0.793
Cardiac manifestation	23 (60.5)	14 (93.3)	0.022
Pulmonary hypertension	13 (34.2)	10 (66.7)	0.063
Ventricular dysfunction	5 (13.5)	9 (60)	0.001
Ventricular hypertrophy	13 (35.1)	6 (40)	0.760
Tricuspid regurgitation	4 (10.8)	6 (42.9)	0.018
Mitral regurgitation	5 (13.5)	2 (13.3)	1.000
Left ventricular outflow tract stenosis	0 (0)	1 (6.7)	0.288
Right ventricular outflow tract stenosis	2 (5.4)	2 (13.3)	0.569

TTTS: twin to twin transfusion syndrome, Values are expressed N (%) or median (interquartile range).

In the multivariate logistic analysis, acute kidney injury (odds ratio [OR] 23.6, 95% confidence interval [95% CI] 2.5–930.3) and cardiac manifestations (OR 28.2, 95% CI 1.3–617.1) were significantly associated with brain injury (Table 4). Among the cardiac manifestations, ventricular dysfunction (OR 22.1, 95% CI 2.4–204.4) and TR (OR 10.6, 95% CI 1.2–96.4) were associated with brain injury in the study population.

**Table 4:** Logistic regression analysis of risk factors for brain injury in TTTS.

	adjOR^a^	95% CI	p	adjOR^b^	95% CI	p
Acute kidney injury	23.6	[1.6, 359.7]	0.023	47.8	[2.5, 930.3]	0.011
Hypotension	5.3	[0.9, 32.2]	0.069	5.6	[0.7, 48.6]	0.118
Cardiac manifestation	28.2	[1.3, 617.1]	0.034			
Ventricular dysfunction				22.1	[2.4, 204.4]	0.006
Tricuspid regurgitation				10.6	[1.2, 96.4]	0.036

^a^adjusted for gestational age, Quintero stage, acute kidney injury, hypotension, cardiac manifestation.

^b^adjusted for gestational age, Quintero stage, acute kidney injury, hypotension, ventricular dysfunction, tricuspid regurgitation.

## Discussion

In the present study, cardiac manifestations immediately after birth were independently associated with perinatal brain injury in premature infants with TTTS, regardless of whether the infant was the donor or recipient. Gestational age at diagnosis of TTTS and Quintero stage were not associated with perinatal brain injury. However, postnatal manifestations implicating perinatal hemodynamic imbalance, such as AKI and hypotension, were associated with perinatal brain injury.

Chronic volume inequality in TTTS can cause both functional and acquired structural cardiac anomalies^11,12^. These changes can start very early in the disease process^13^ and may be demonstrated in the earliest stage of TTTS^11^, even before TTTS is diagnosed^14^. For the recipient, the preload volume is increased, with significantly higher cardiac output^15^, and the afterload is increased in the form of increased resistance and fetal hypertension, attributed to the presence of vasoactive mediators such as endothelin^16^. Eventually, decreased forward blood flow through the right side of the heart can cause right ventricular outflow track obstruction (RVOTO)^17^. For the donor, decreased blood volume results in diminished left-side cardiac output, and hypoperfusion stimulates upregulation of the renin-angiotensin system to attempt to maintain perfusion, which results in increased vascular resistance with smooth muscle hypertrophy and may lead to intrauterine growth restriction, cerebral redistribution and abnormal arterial Doppler assessment^18^. In contrast to recipient twins, acquired cardiac manifestations of the donor twin are rare. In our study, there were cardiac manifestations in 47.8% of donor twins and 86.7% of recipient twins. Severe forms of cardiac involvement, such as outflow tract stenosis, occurred only in the recipient twins (13.8%).

Brain injuries in twin pregnancy are attributable to hemodynamic and hematological disorders; 30~60% of brain injuries occur during the antenatal period^8,19,20^. During fetal life, hemodynamic instability may trigger both high flow lesions and low flow lesions that occur in utero as a result of ischemic or hemorrhagic events^21,22^. A retrospective study of TTTS with serial echocardiography reported that diastolic dysfunction and cerebroplacental redistribution precede findings of overt cardiomyopathy in recipient twins with early stage TTTS. This might contribute to brain injury despite the successful treatment of TTTS^23^. In the present study, brain injury was not associated with treatment outcomes or the timing of treatment. Rather, immediate cardiac manifestations, as surrogate markers for acute or chronic hemodynamic disturbances of TTTS during the fetal period, especially ventricular dysfunction and TR, were associated with brain injury.

Furthermore, morbidities in the immediate postnatal period reflecting hemodynamic disturbances in the fetal period were analyzed in the present study. Newborns with TTTS may experience hemodynamic instability after birth; 30~70% of brain injuries occur during the postnatal period^5,24^. In the present study, one-third of the study population experienced hypotension during the first week of life, and 17% of the study population had AKI. Interestingly, AKI was associated with brain injury, and postnatal hypotension tended to be associated with brain injury with borderline significance.

The mechanism of decreased renal function in the donor is thought to be secondary glomerular and tubular damage due to hypoxic-ischemic injury and chronic renal parenchymal deterioration^2^. AKI was observed in the recipients in this study, which is consistent with previous studies^25^. In our study, Quintero stage was not associated with brain injury. Recent studies have reported that a higher Quintero stage was not a risk factor for cerebral injury after fetal laser surgery^8,26^. Although staging is based on abnormal Doppler findings, it does not quantify the cardiac involvement of TTTS. The Children’s Hospital of Philadelphia (CHOP) cardiovascular score includes several fetal cardiac findings, and one study shows that the CHOP stage is more accurate than the Quintero stage for predicting brain injury or neurodevelopmental outcomes in TTTS. It aims to systematically and objectively calculate hemodynamic changes in the fetus^17^.

This study has certain limitations. It was retrospectively designed at a single center, and we included patients who were admitted to the NICU. Newborns who were born after 35 weeks of gestation, weighed at least 1,800 grams at birth and were healthy in the delivery room are not admitted to the NICU at our hospital. We speculated that there was an association between postnatal cardiac manifestations and brain injury among survivors of TTTS; therefore, other important pregnancy outcomes of TTTS, such as fetal demise, were not considered in our study. Moreover, fetal echocardiography and fetal brain imaging were not analyzed in this study because they were not conducted in the course of routine antenatal care.

In conclusion, this is the first study to report that immediate postnatal cardiac manifestations, such as ventricular dysfunction and TR, and early postnatal systemic signs implicating hemodynamic disturbances, such as AKI and postnatal hypotension, are associated with perinatal brain injury of TTTS. Further studies including fetal echocardiac findings and fetal brain imaging might elucidate this association more clearly.

## Materials and Methods

Maternal and neonatal medical records of preterm infants with TTTS who were born at <35 weeks of gestation and were admitted to the neonatal intensive care unit at Seoul National University Children’s Hospital between January 2011 and January 2018 were reviewed. TTTS was prenatally diagnosed using antenatal ultrasound and staged according to the Quintero criteria^27^. The highest stage of TTTS during pregnancy was analyzed irrespective of treatment modalities and responses.

Data pertaining to gestational age at diagnosis of TTTS, gestational age at delivery, TTTS stage at treatment, and the treatment method were collected. Birth weight, hemoglobin level at birth, and serum creatinine level during the first week were reviewed. First brain ultrasounds (US) were performed within 24 hours after birth by experienced pediatric radiologists who were blinded to the patient details and thereafter according to the clinical protocol (one or two more times by end of the first week, followed by at least once every two weeks). Brain MRI was performed within 7 days after birth when serious hypoxic insult was suspected on brain US. The first postnatal echocardiography was performed within 48 hours after birth by experienced pediatric cardiologists blinded to the clinical information.

Hypotension was defined as low blood pressure for which inotropic medication was given during the first week. Treatment failure was defined as disease progression or the need for further treatment despite amnioreduction or fetoscopic laser coagulation. Postnatal morbidities, including RDS, BPD, NEC, PDA, sepsis, and retinopathy of prematurity (ROP), were also reviewed. BPD was diagnosed according to the National Institute of Child Health and Human Development (NICHD) definition^28^. NEC was diagnosed according to the modified Bell’s criteria (≥stage 2)^29^. AKI was defined as a serum creatinine level of >1.5 mg/dL for more than 48 hours or the presence of oliguria (urine output <1 mL/kg/h)^30^.

Brain injury was categorized during the neonatal period as hypoxic-ischemic encephalopathy (HIE), severe intraventricular hemorrhage (IVH grades 3 and 4), ventricular dilatation, ischemic or hemorrhagic stroke, and periventricular leukomalacia (PVL). IVH was classified according to Papile *et al*.^31^. Postnatal echocardiographic findings of mitral regurgitation, ventricular dysfunction, ventricular hypertrophy, LVOT stenosis, or RVOT stenosis and findings of pulmonary hypertension, such as right to left shunt, TR (velocity >3 m/s), and septal deviation, were reviewed.

Analysis was performed with the SPSS statistical package 19.0. Differences between categorical variables were analyzed using the chi-square test or Fisher’s exact test. Differences between continuous variables were tested using an independent samples t-test. Multivariate logistic regression analysis was conducted to define the risk factors for brain injury in the study population, adjusted by gestational age, Quintero stage, AKI, hypotension and cardiac manifestation in the first model. In the second model, cardiac manifestation was further defined as ventricular dysfunction and tricuspid regurgitation during multivariate logistic regression analysis. A statistically significant difference was defined as p < 0.05. The study was approved by the Institutional Review Board of Seoul National University Hospital. The need for informed consent was waived by the institutional review board because of the nature of the retrospective study. All methods used in this study were performed in accordance with the relevant guidelines and regulations.
